# Supplementary material for: A Likelihood Approach for Real-Time Calibration of Stochastic Compartmental Epidemic Models
Source: PLoS Comput Biol. 2017 Jan 17;13(1):e1005257. doi: 10.1371/journal.pcbi.1005257 (PMC5240920; doi:10.1371/journal.pcbi.1005257)
Supplement: S1 File — (TAR.GZ) [file pcbi.1005257.s014.tar.gz › HSPH_Online-SI-Revision/MSS10/n100-mild/n100-mild_NEW_table-8.pdf]

1	R0	{1.24125, 3.38982, 0.1}	{1.23243, 3.03411, 0.02}	{{1.22544, 2.5364, 0.}, {1.25474, 3.26445, 0.}}	{1.57614, 11.7644, 116.}	inf	218.	187.868	{111.277, 268.928}	27.2168	0.152843	newly	544.	554.03	{296., 798.}	29.7629	0.14006	Reff	1.2258	1.21746	{1.20942, 1.23983}	1.57238	ar	34 926.	34 876.5	{32 824., 38 136.}	6.60754
2	R0	{1.29554, 4.51878, 0.1}	{1.24525, 2.8704, 0.}	{{1.22544, 2.5364, 0.}, {1.25474, 3.26445, 0.}}	{3.88171, 36.4783, 100.}	inf	496.	361.827	{231.072, 473.183}	27.4269	0.154292	newly	1039.	1171.77	{700., 1616.}	26.7729	0.106544	Reff	1.26727	1.21975	{1.20011, 1.22885}	3.74998	ar	42 828.	36 439.7	{34 110., 38 302.}	14.9163
3	R0	{1.2443, 3.64292, 0.1}	{1.24176, 3.01242, 0.}	{{1.22544, 2.5364, 0.}, {1.25474, 3.26445, 0.}}	{1.00894, 17.6833, 100.}	inf	288.	275.006	{171.236, 392.864}	25.786	0.131852	newly	581.	836.44	{523., 1140.}	51.5594	0.180919	Reff	1.22536	1.22302	{1.20622, 1.23602}	1.00695	ar	37 087.	36 138.8	{33 294., 38 442.}	4.68881
4	R0	{1.38465, 3.43331, 0.1}	{2.30571, 4.41272, 0.08}	{{1.67287, 2.12856, 0.}, {2.8702, 6.87793, 0.}}	{66.5195, 62.88, 164.}	inf	73.	162.869	{30.6918, 281.406}	145.708	0.402834	newly	229.	1136.47	{347., 2010.}	400.135	0.56945	Reff	1.38185	2.2986	{1.66844, 2.86101}	66.3417	ar	49 511.	81 826.1	{67 670., 93 142.}	65.2685
5	R0	{1.25409, 4.1068, 0.1}	{1.22335, 2.80306, 0.02}	{{1.22544, 2.5364, 0.}, {1.25474, 3.26445, 0.}}	{2.46383, 31.7458, 118.}	inf	293.	155.425	{53.5829, 248.247}	47.8148	0.33834	newly	618.	508.68	{183., 871.}	37.0971	0.209953	Reff	1.23932	1.21061	{1.21157, 1.23981}	2.35363	ar	38 571.	33 584.1	{31 579., 37 428.}	12.9911
6	R0	{1.28821, 4.2016, 0.1}	{1.2425, 2.69397, 0.}	{{1.22544, 2.5364, 0.}, {1.24751, 3.07157, 0.}}	{3.54804, 35.8824, 100.}	inf	415.	298.327	{172.565, 439.737}	32.0277	0.190925	newly	847.	1034.15	{539., 1545.}	37.1464	0.142433	Reff	1.26157	1.21826	{1.2005, 1.22521}	3.43292	ar	41 468.	35 947.	{33 744., 38 117.}	13.314
7	R0	{1.24369, 2.56007, 0.1}	{1.53302, 3.94666, 0.11}	{{1.24751, 2.5364, 0.}, {1.74092, 5.58477, 0.}}	{23.2932, 58.5721, 196.}	inf	56.	112.082	{49.3323, 174.968}	108.652	0.305643	newly	202.	362.39	{170., 640.}	89.3119	0.249598	Reff	1.24058	1.52826	{1.24431, 1.73509}	23.2185	ar	38 603.	58 026.	{37 290., 71 095.}	51.7758
8	R0	{1.232, 3.53942, 0.1}	{1.24086, 2.56548, 0.}	{{1.24751, 2.5364, 0.}, {1.24751, 2.5364, 0.}}	{1.58455, 27.517, 100.}	inf	337.	242.652	{124.354, 354.792}	31.7087	0.212997	newly	787.	902.88	{430., 1343.}	38.2745	0.180195	Reff	1.2117	1.22157	{1.22545, 1.22961}	1.66785	ar	34 555.	35 826.	{34 880., 38 016.}	7.373
9	R0	{1.36262, 3.85631, 0.1}	{1.48714, 3.99203, 0.02}	{{1.24751, 2.5364, 0.}, {1.53501, 4.06661, 0.}}	{11.3237, 13.2139, 118.}	inf	149.	150.525	{79.6128, 204.804}	29.5169	0.140257	newly	418.	415.29	{212., 560.}	27.7488	0.131808	Reff	1.35645	1.48032	{1.24306, 1.52993}	11.306	ar	48 410.	55 857.6	{37 001., 61 389.}	23.0472
10	R0	{1.28702, 4.67968, 1.}	{1.06733, 2.19577, 0.01}	{{1.06738, 2.19254, 0.}, {1.06738, 2.19254, 0.}}	{17.0699, 53.0787, 99.}	inf	588.	230.445	{107.602, 380.799}	60.8087	0.463132	newly	1085.	776.18	{339., 1286.}	35.2737	0.233074	Reff	1.24853	1.03922	{1.0376, 1.04058}	16.7642	ar	41 092.	12 980.1	{10 261., 15 771.}	68.4122
11	R0	{1.35752, 3.47129, 0.1}	{2.32554, 14.6127, 2.55}	{{1.25474, 3.26445, 0.}, {2.85956, 19.8999, 3.}}	{73.4944, 323.974, 2480.}	inf	93.	536.814	{133.369, 754.42}	483.639	0.729947	newly	286.	343.27	{291., 406.}	26.458	0.111506	Reff	1.35065	2.3025	{1.24862, 2.82539}	72.6639	ar	44 669.	81 586.	{38 083., 93 015.}	87.1495
12	R0	{1.35929, 5.83647, 0.1}	{1.23692, 3.18316, 0.02}	{{1.22544, 3.07157, 0.}, {1.25474, 3.26445, 0.}}	{9.00222, 45.4609, 116.}	inf	673.	406.755	{293.136, 524.698}	39.5609	0.231965	newly	1048.	1118.24	{777., 1382.}	18.6756	0.0808267	Reff	1.3216	1.20591	{1.19377, 1.22445}	8.75332	ar	46 715.	35 407.2	{32 820., 38 463.}	24.206
13	R0	{1.28019, 3.29908, 0.1}	{1.44586, 3.64234, 0.01}	{{1.24751, 2.5364, 0.}, {1.53501, 4.02511, 0.}}	{14.0473, 21.059, 108.}	inf	93.	115.813	{46.2698, 170.686}	45.4547	0.186254	newly	305.	351.1	{151., 521.}	40.577	0.179259	Reff	1.27548	1.44018	{1.24278, 1.52904}	14.0174	ar	40 941.	53 480.	{36 804., 60 941.}	34.9421
14	R0	{1.36661, 3.48318, 0.1}	{1.43044, 2.3959, 0.19}	{{1.24751, 1.89883, 0.}, {1.65456, 3.37285, 1.}}	{6.58819, 39.7498, 264.}	inf	101.	96.4416	{21.6708, 174.83}	49.1035	0.272201	newly	296.	653.43	{129., 1358.}	144.139	0.36394	Reff	1.36217	1.42581	{1.24446, 1.6491}	6.58075	ar	48 672.	51 986.3	{37 577., 66 516.}	12.226
15	R0	{1.35865, 3.84123, 0.1}	{1.27173, 2.54283, 0.02}	{{1.24751, 2.5364, 0.}, {1.39162, 2.5364, 0.}}	{7.67206, 34.0048, 116.}	inf	112.	109.94	{29.1461, 174.957}	40.5038	0.216738	newly	273.	479.47	{120., 766.}	97.5348	0.289219	Reff	1.35217	1.26568	{1.24084, 1.38469}	7.6612	ar	48 598.	39 091.4	{35 644., 50 696.}	22.0142
16	R0	{1.22879, 3.98329, 0.1}	{1.23977, 2.80529, 0.}	{{1.22544, 2.5364, 0.}, {1.24751, 3.07157, 0.}}	{1.10131, 29.5735, 100.}	inf	457.	367.653	{258.164, 495.341}	23.095	0.128811	newly	913.	1218.4	{795., 1677.}	40.9091	0.146374	Reff	1.20087	1.21271	{1.19787, 1.22351}	1.12603	ar	34 405.	35 756.5	{33 682., 37 909.}	5.03369
17	R0	{1.34925, 3.64524, 0.1}	{1.32441, 3.37509, 0.22}	{{1.22544, 2.5364, 0.}, {1.44762, 3.83841, 0.}}	{12.9681, 42.5951, 308.}	inf	135.	117.948	{16.8129, 175.056}	54.4498	0.327985	newly	291.	339.38	{63., 621.}	52.7491	0.272954	Reff	1.34265	1.31783	{1.22008, 1.44117}	12.8893	ar	46 871.	39 197.3	{34 303., 54 764.}	29.1197
18	R0	{1.33054, 2.25275, 0.1}	{2.31105, 3.01424, 0.}	{{1.67287, 1.89883, 0.}, {2.90743, 4.40915, 0.}}	{73.6928, 39.3474, 100.}	inf	11.	35.0046	{9.63204, 62.4809}	226.522	0.463253	newly	52.	689.67	{134., 1389.}	1229.13	0.949573	Reff	1.33009	2.30967	{1.672, 2.90581}	73.6482	ar	44 336.	82 538.9	{67 696., 93 435.}	86.1668
19	R0	{1.28394, 3.69859, 0.1}	{1.14342, 2.59583, 0.04}	{{1.06738, 2.19254, 0.}, {1.24751, 3.07157, 0.}}	{10.9474, 29.9707, 136.}	inf	236.	184.912	{61.9578, 300.946}	35.5487	0.259073	newly	569.	575.66	{210., 877.}	37.1916	0.214556	Reff	1.26314	1.12545	{1.05011, 1.22838}	10.9036	ar	39 558.	22 763.9	{10 361., 35 603.}	42.4728
20	R0	{1.2883, 2.26493, 0.1}	{1.99768, 5.27847, 0.22}	{{1.24751, 2.5364, 0.}, {2.8702, 7.22215, 1.}}	{56.8092, 133.581, 292.}	inf	9.	9.64856	{2.41547, 19.232}	57.419	0.272671	newly	36.	41.71	{5., 80.}	89.75	0.39379	Reff	1.28811	1.99735	{1.24741, 2.86955}	56.8055	ar	40 768.	67 183.7	{80., 92 957.}	87.8698
21	R0	{1.34098, 2.78621, 0.1}	{2.14371, 3.4395, 0.07}	{{1.39162, 1.89883, 0.}, {2.96265, 4.48882, 0.}}	{62.0191, 38.9064, 158.}	inf	10.	17.7166	{4.12465, 32.7655}	100.843	0.310536	newly	66.	185.44	{19., 391.}	214.091	0.465451	Reff	1.34068	2.14304	{1.39118, 2.9618}	62.0041	ar	44 605.	72 639.9	{34 825., 93 844.}	79.6384
22	R0	{1.33783, 5.95911, 0.1}	{1.22624, 3.08602, 0.01}	{{1.22544, 3.07157, 0.}, {1.25474, 3.26445, 0.}}	{8.34115, 48.2134, 108.}	inf	837.	403.811	{249.636, 553.59}	51.755	0.334459	newly	1123.	1141.62	{780., 1496.}	22.1158	0.0981606	Reff	1.29617	1.19336	{1.19069, 1.21989}	7.93204	ar	45 418.	34 177.1	{32 693., 36 825.}	24.7498
23	R0	{1.33359, 3.3069, 0.1}	{2.50357, 14.6126, 2.78}	{{2.41896, 13.1237, 2.}, {2.688, 15.191, 3.}}	{87.7313, 341.882, 2680.}	inf	97.	489.332	{420.003, 553.574}	404.466	0.700498	newly	260.	338.97	{290., 385.}	30.3731	0.112496	Reff	1.32804	2.4833	{2.39974, 2.66433}	86.9893	ar	46 245.	89 260.7	{88 134., 91 362.}	93.0169
24	R0	{1.30588, 2.56123, 0.1}	{2.30818, 3.54061, 0.08}	{{1.39162, 1.89883, 0.}, {2.99281, 5.42517, 0.}}	{76.753, 52.0606, 164.}	inf	22.	33.1299	{5.9998, 62.7918}	86.1596	0.31613	newly	124.	353.87	{55., 649.}	207.734	0.445993	Reff	1.30531	2.30684	{1.39116, 2.99149}	76.7281	ar	42 383.	80 720.9	{50 587., 94 019.}	90.4559
25	R0	{1.29813, 4.51849, 0.1}	{1.10448, 2.43738, 0.15}	{{1.06738, 2.19254, 0.}, {1.22544, 3.07157, 1.}}	{14.9178, 46.0577, 226.}	inf	361.	211.555	{96.4493, 322.233}	42.8353	0.294975	newly	633.	685.08	{318., 1039.}	36.5182	0.168415	Reff	1.27199	1.08385	{1.04673, 1.20125}	14.7907	ar	43 371.	18 007.9	{9557., 34 512.}	58.4795
26	R0	{1.33231, 3.99953, 0.1}	{1.08026, 2.10952, 0.56}	{{1.06738, 1.81005, 0.}, {1.06836, 2.19254, 2.}}	{18.9182, 47.2559, 604.}	inf	222.	134.66	{62.7954, 216.744}	42.5362	0.28965	newly	479.	492.98	{214., 834.}	39.2443	0.192199	Reff	1.3147	1.06692	{1.05352, 1.05588}	18.8467	ar	44 733.	14 759.7	{10 663., 16 267.}	67.0049
27	R0	{1.30555, 4.61445, 0.1}	{1.24071, 2.7139, 0.02}	{{1.22544, 2.5364, 0.}, {1.24751, 3.07157, 0.}}	{4.96662, 41.187, 118.}	inf	301.	215.633	{112.702, 306.861}	31.5153	0.190059	newly	599.	754.42	{390., 1103.}	43.5092	0.160793	Reff	1.2885	1.22556	{1.20991, 1.23397}	4.88444	ar	42 624.	36 083.8	{34 091., 38 129.}	15.3439
28	R0	{1.26819, 5.34969, 0.1}	{1.2445, 3.20222, 0.}	{{1.22544, 3.07157, 0.}, {1.25474, 3.26445, 0.}}	{1.89584, 40.142, 100.}	inf	559.	342.914	{216.15, 452.443}	38.8822	0.232946	newly	884.	956.23	{614., 1232.}	25.1505	0.108331	Reff	1.2407	1.22021	{1.20112, 1.23129}	1.68177	ar	38 946.	36 344.5	{33 360., 38 669.}	6.91717
29	R0	{1.19925, 3.00394, 0.1}	{1.23978, 2.50978, 0.07}	{{1.24751, 2.5364, 0.}, {1.24751, 2.5364, 0.}}	{4.26693, 16.4503, 164.}	inf	185.	194.05	{82.7271, 296.089}	35.0123	0.165201	newly	516.	742.69	{319., 1172.}	59.9981	0.21206	Reff	1.18521	1.22514	{1.2306, 1.23397}	4.25314	ar	31 727.	35 531.2	{34 703., 37 938.}	17.1177
30	R0	{1.35749, 3.75483, 0.1}	{1.22087, 3.16066, 0.09}	{{1.06738, 2.19254, 0.}, {1.28596, 3.98513, 0.}}	{10.1971, 18.6766, 176.}	inf	91.	56.4097	{12.0442, 100.217}	46.3178	0.362154	newly	259.	161.12	{22., 318.}	49.112	0.388479	Reff	1.35287	1.21713	{1.0643, 1.28202}	10.1656	ar	48 244.	31 805.2	{11 569., 40 743.}	34.3164
31	R0	{1.27131, 4.2161, 0.1}	{1.17143, 2.66623, 0.04}	{{1.06738, 2.19254, 0.}, {1.24751, 3.07157, 0.}}	{7.85668, 36.7609, 136.}	inf	214.	126.57	{62.2702, 204.411}	43.315	0.293119	newly	451.	409.6	{192., 610.}	33.1619	0.179536	Reff	1.25808	1.16024	{1.05717, 1.2364}	7.77649	ar	38 299.	26 483.4	{10 237., 37 261.}	31.014
32	R0	{1.31827, 6.18119, 0.1}	{1.23307, 3.01295, 0.}	{{1.22544, 2.5364, 0.}, {1.25474, 3.26445, 0.}}	{6.46291, 51.2562, 100.}	inf	869.	482.701	{364.784, 594.843}	44.4533	0.262719	newly	1107.	1420.48	{1071., 1726.}	30.0578	0.109175	Reff	1.27462	1.19701	{1.18855, 1.21663}	6.08921	ar	45 262.	35 067.5	{32 919., 37 439.}	22.5234
33	R0	{1.20398, 1.35167, 0.1}	{2.33083, 3.41662, 0.}	{{1.67287, 1.89883, 0.}, {2.99949, 5.42517, 0.}}	{93.5941, 152.77, 100.}	inf	3.	25.7367	{5.61869, 49.8822}	759.398	0.821165	newly	20.	422.	{41., 645.}	2013.4	1.03026	Reff	1.20375	2.32981	{1.67218, 2.99769}	93.5456	ar	33 329.	81 468.5	{67 463., 94 016.}	146.435
34	R0	{1.32776, 3.146, 0.1}	{1.31467, 2.81831, 0.07}	{{1.24751, 2.5364, 0.}, {1.47608, 3.83841, 0.}}	{8.26754, 24.4866, 160.}	inf	86.	88.1382	{25.8092, 147.453}	45.018	0.226771	newly	292.	350.22	{98., 589.}	56.2534	0.233131	Reff	1.32356	1.31044	{1.2429, 1.47137}	8.25121	ar	44 872.	41 946.1	{35 771., 56 455.}	20.4234
35	R0	{1.279, 1.11336, 0.1}	{2.30697, 2.76954, 0.}	{{1.90958, 1.89883, 0.}, {2.90743, 4.10463, 0.}}	{80.3727, 148.755, 100.}	inf	8.	51.4012	{6.65223, 93.2519}	550.315	0.713264	newly	51.	1221.82	{156., 2156.}	2299.88	1.19267	Reff	1.27844	2.30488	{1.90791, 2.90382}	80.289	ar	41 033.	83 075.1	{69 706., 93 349.}	104.457
36	R0	{1.20029, 1.94717, 0.1}	{1.9414, 4.09922, 0.13}	{{1.22544, 2.19254, 0.}, {2.82914, 6.2492, 0.}}	{63.1429, 110.522, 214.}	inf	9.	11.6902	{2.67454, 22.3164}	67.4548	0.274836	newly	29.	90.01	{9., 234.}	243.414	0.397553	Reff	1.20009	1.941	{1.22519, 2.8286}	63.1372	ar	33 234.	63 517.7	{40., 92 773.}	118.523
37	R0	{1.33216, 3.69685, 0.1}	{1.38858, 1.88257, 0.09}	{{1.39162, 1.89883, 0.}, {1.39162, 1.89883, 0.}}	{4.42424, 49.0764, 184.}	inf	191.	196.846	{29.9183, 401.567}	58.2407	0.318677	newly	501.	1563.23	{227., 3105.}	228.92	0.477902	Reff	1.3191	1.3749	{1.37484, 1.38013}	4.4281	ar	45 235.	49 980.6	{48 873., 51 219.}	10.8788
38	R0	{1.25434, 2.04314, 0.1}	{2.18354, 2.79012, 0.02}	{{1.39162, 1.89883, 0.}, {2.90743, 4.18259, 0.}}	{74.0789, 38.7044, 116.}	inf	23.	30.1739	{6.74989, 58.8688}	68.4295	0.282387	newly	145.	606.67	{95., 1123.}	331.	0.493281	Reff	1.25374	2.18229	{1.39112, 2.90568}	74.0628	ar	38 662.	78 054.6	{50 843., 93 393.}	103.888
39	R0	{1.27466, 3.0741, 0.1}	{1.40018, 1.93768, 0.01}	{{1.39162, 1.89883, 0.}, {1.39162, 1.89883, 0.}}	{9.84701, 37.3563, 108.}	inf	125.	150.563	{33.5444, 281.771}	65.6436	0.308994	newly	445.	1220.8	{228., 2300.}	194.782	0.444273	Reff	1.2688	1.39337	{1.38304, 1.38676}	9.81848	ar	40 181.	50 990.2	{49 479., 51 846.}	26.9013
40	R0	{1.33045, 2.68154, 0.1}	{1.47345, 1.98759, 0.}	{{1.39162, 1.89883, 0.}, {1.70301, 2.24898, 0.}}	{10.8732, 25.8787, 100.}	inf	25.	74.4708	{19.967, 136.585}	208.529	0.444908	newly	85.	687.81	{144., 1283.}	714.6	0.814402	Reff	1.32907	1.47118	{1.38876, 1.69937}	10.818	ar	45 951.	54 923.3	{49 611., 69 088.}	21.9436
41	R0	{1.37003, 2.83984, 0.1}	{2.07499, 3.40882, 0.02}	{{1.24751, 2.15811, 0.}, {2.96265, 4.40915, 0.}}	{55.8431, 34.9444, 116.}	inf	9.	15.4122	{3.04087, 30.2567}	102.667	0.313617	newly	52.	146.69	{9., 341.}	222.827	0.504383	Reff	1.36976	2.0744	{1.24726, 2.96053}	55.8302	ar	48 926.	70 350.	{35 399., 93 660.}	64.6905
42	R0	{1.3003, 2.94885, 0.1}	{1.74535, 1.89063, 0.42}	{{1.42206, 1.31573, 0.}, {2.01676, 2.15811, 1.}}	{34.227, 45.2998, 440.}	inf	51.	95.4904	{25.5585, 179.643}	109.917	0.330981	newly	214.	2272.25	{461., 4864.}	966.201	0.898154	Reff	1.29797	1.7414	{1.41881, 2.01378}	34.163	ar	44 237.	67 646.9	{52 426., 80 308.}	54.9123
43	R0	{1.33756, 2.92653, 0.1}	{2.34212, 3.19754, 0.01}	{{1.67287, 2.12856, 0.}, {2.96265, 4.40915, 0.}}	{75.104, 33.8027, 108.}	inf	19.	27.844	{8.18379, 48.9214}	74.4512	0.267624	newly	61.	479.94	{79., 919.}	692.918	0.720927	Reff	1.33705	2.34099	{1.67238, 2.96138}	75.0862	ar	47 342.	82 446.9	{68 115., 93 743.}	76.1499
44	R0	{1.35801, 2.92276, 0.1}	{1.57161, 3.93106, 0.06}	{{1.22544, 2.19254, 0.}, {2.14326, 5.68021, 0.}}	{22.8276, 42.797, 150.}	inf	11.	24.5184	{4.5249, 43.8455}	144.443	0.382333	newly	66.	81.28	{17., 164.}	70.7273	0.296441	Reff	1.35749	1.57077	{1.22487, 2.14201}	22.8124	ar	47 940.	53 607.6	{14 740., 83 436.}	42.8076
45	R0	{1.31342, 4.02649, 0.1}	{1.43777, 3.84789, 0.04}	{{1.24751, 2.5364, 0.}, {1.47608, 4.06661, 0.}}	{11.3077, 10.3342, 138.}	inf	222.	299.106	{187.293, 411.071}	42.6579	0.149554	newly	477.	820.14	{530., 1111.}	74.1593	0.228415	Reff	1.30142	1.42342	{1.23637, 1.46173}	11.2093	ar	42 995.	52 827.9	{37 083., 57 235.}	28.0509
46	R0	{1.37919, 3.75889, 0.1}	{2.0904, 10.6166, 2.55}	{{1.42206, 1.31573, 1.}, {2.79269, 19.984, 4.}}	{52.018, 241.694, 2456.}	inf	78.	226.66	{24.5614, 404.374}	219.842	0.501739	newly	193.	747.67	{143., 2081.}	302.306	0.36754	Reff	1.37534	2.08048	{1.41776, 2.77657}	51.7203	ar	49 157.	72 382.5	{52 298., 92 549.}	50.836
47	R0	{1.37458, 3.56117, 0.1}	{1.76901, 4.64182, 0.32}	{{1.39162, 1.89883, 0.}, {2.61341, 12.7047, 1.}}	{28.6943, 65.4585, 376.}	inf	90.	128.78	{37.2093, 258.81}	70.0466	0.241877	newly	236.	489.71	{182., 882.}	119.064	0.293793	Reff	1.37118	1.76369	{1.38822, 2.60209}	28.6253	ar	48 478.	66 913.	{50 171., 90 855.}	38.0276
48	R0	{1.33393, 3.78296, 0.1}	{1.24689, 3.38111, 0.05}	{{1.22544, 3.07157, 0.}, {1.28596, 3.98513, 0.}}	{6.69518, 13.7333, 140.}	inf	172.	157.112	{93.2965, 225.495}	26.3657	0.138103	newly	441.	417.56	{253., 568.}	25.0658	0.124859	Reff	1.3221	1.23601	{1.21413, 1.27446}	6.67964	ar	44 885.	36 205.	{32 508., 40 946.}	19.7521
49	R0	{1.24918, 2.39046, 0.1}	{1.42301, 1.32421, 1.01}	{{1.42206, 1.31573, 1.}, {1.42206, 1.31573, 1.}}	{13.9157, 44.6042, 912.}	inf	41.	83.567	{23.9198, 155.517}	122.359	0.360699	newly	241.	1592.38	{437., 2839.}	567.353	0.765554	Reff	1.2448	1.41741	{1.41538, 1.41731}	13.867	ar	35 969.	52 166.8	{51 588., 53 451.}	47.0153
50	R0	{1.28281, 2.3032, 0.1}	{1.40318, 1.90922, 0.}	{{1.39162, 1.89883, 0.}, {1.39162, 1.89883, 0.}}	{9.38298, 17.1056, 100.}	inf	28.	109.509	{28.1633, 203.864}	297.96	0.542662	newly	129.	905.53	{243., 1714.}	605.574	0.7668	Reff	1.28046	1.39946	{1.38661, 1.3891}	9.29359	ar	40 223.	51 272.7	{49 660., 51 653.}	27.471
